# Editorial: Neuroepidemiology of stroke in low and middle income countries

**DOI:** 10.3389/fneur.2022.1059974

**Published:** 2022-11-08

**Authors:** Tapas Kumar Banerjee

**Affiliations:** National Neurosciences Centre Calcutta, Kolkata, India

**Keywords:** stroke, epidemiology, incidence, mortality, opium, Moya-Moya angiopathy

Since 1990 there has been a decline in the stroke-related incidence and mortality in the high-income countries (HIC). On the contrary, in low and middle-income countries (LMIC) the incidence and mortality rates of stroke have not shown a tangible improvement and their absolute numbers have increased. Currently, LMIC account for >75% of global stroke related deaths and >80% of disability-adjusted life-years (DALY) (1). A comprehensive epidemiological data on cerebrovascular disease from LMIC is, therefore, extremely valuable and the need of the hour. In this research collection, we have compiled original articles from several countries of Asia, Africa and South America that brought out certain important facets of stroke epidemiology.

Data derived from the African nations, namely, Ethiopia and Zanzibar (Jørgensen et al.) revealed younger age of stroke onset (median age 62 years) and higher proportion of cerebral hemorrhage (around 30%) in their subjects compared to those from the Western countries. The age-standardized incidence rate of stroke, as derived from the Zanzibar study was much higher than that from HIC. The study also underscores the urgent need for enhancing public awareness in taking regular medications for hypertension and diabetes mellitus, the two common risk factors of stroke.

China is a high middle-income country with the second largest economy in the world. But yet the age-standardized incidence of stroke in China has remained very high (2). China alone accounts for one-third of world's stroke mortality. A careful nationwide 4-year prospective cohort Chinese study indicated that the combined effect of elevated C-reactive protein and hypertension increase the risk of stroke manifold among the middle-aged and the elderly, perhaps by accelerating vascular inflammation and atherogenesis.

Many countries have 2-tier healthcare system: one that is government-provided universal healthcare and is essentially free for all; the other privately-funded for those who can afford. In several middle- income countries (MIC), some privately funded healthcare provides very advanced quality of treatment on a par with the best in the world but for that the beneficiary has to pay a substantial amount from their own pocket. This results in clear disparity in terms of medical outcome between the two tiers. The government should aim to close this gap in order to deliver universal good quality healthcare. In a study from Brazil, stroke outcomes were compared between those treated in a good quality large public medical facility and those managed in a private hospital. Although appropriate medical treatment including intravenous thrombolysis were administered to the patients in both the hospitals, worse outcome was demonstrated in the former group. Among other factors, paucity of post-stroke rehabilitation program in public hospital is responsible (Martins et al.).

Earlier, the INTERSTROKE study did show that the principal risk factors for stroke include hypertension, diabetes mellitus, smoking and physical inactivity (3). Data from our research papers upheld the above observation. Analysis of young stroke cases in Saudi Arabia, however, revealed dyslipidemia the commonest risk factor (Eltemamy et al.). On the other hand, opium dependence is an important risk factor for stroke in Iran. This is because opium abuse in Iran and Afghanistan is 3 times higher than global average. Opium leads to atherogenesis and also enhances the propensity for diabetes and hypertension.

Moya-Moya angiopathy (MMA) is a rare cause of stroke, more often seen in pediatric population. It is far more common among the Orientals than among Whites. So far there had been no large clinical series on MMA from India. The interesting study from Kolkata, the Eastern Indian metropolis, presented the largest series of angiographically proven MMA (Das et al.). Prevalence of posterior circulation MMA in India was found to be 2 times higher than the other Asian countries and 6 times higher than the Western countries.

## Author contributions

The author confirms being the sole contributor of this work and has approved it for publication.

## Conflict of interest

The author declares that the research was conducted in the absence of any commercial or financial relationships that could be construed as a potential conflict of interest.

## Publisher's note

All claims expressed in this article are solely those of the authors and do not necessarily represent those of their affiliated organizations, or those of the publisher, the editors and the reviewers. Any product that may be evaluated in this article, or claim that may be made by its manufacturer, is not guaranteed or endorsed by the publisher.
